# Genome-scale metabolic models reveal determinants of phenotypic differences in non-*Saccharomyces* yeasts

**DOI:** 10.1186/s12859-023-05506-7

**Published:** 2023-11-21

**Authors:** Jakob P. Pettersen, Sandra Castillo, Paula Jouhten, Eivind Almaas

**Affiliations:** 1 Department of Biotechnology and Food Science, NTNU-Norwegian University of Science and Technology, Trondheim, Norway; 2 Department of Public Health and General Practice, K.G. Jebsen Center for Genetic Epidemiology, NTNU- Norwegian University of Science and Technology, Trondheim, Norway; 3 VTT Technical Research Centre of Finland, Espoo, Finland; 4 Department of Bioproducts and Biosystems, Aalto University, Espoo, Finland

**Keywords:** *Metschnikowia pulcherrima*, sMOMENT, Genome-scale models, Electron transport chain, Complex I, Yeast, Metabolic modelling, Automated reconstructions, Enzymatic constraints, Alternative pathways, decFBA

## Abstract

**Background:**

Use of alternative non-*Saccharomyces* yeasts in wine and beer brewing has gained more attention the recent years. This is both due to the desire to obtain a wider variety of flavours in the product and to reduce the final alcohol content. Given the metabolic differences between the yeast species, we wanted to account for some of the differences by using *in silico* models.

**Results:**

We created and studied genome-scale metabolic models of five different non-*Saccharomyces* species using an automated processes. These were: *Metschnikowia pulcherrima*, *Lachancea thermotolerans*, *Hanseniaspora osmophila*, *Torulaspora delbrueckii* and *Kluyveromyces lactis*. Using the models, we predicted that *M. pulcherrima*, when compared to the other species, conducts more respiration and thus produces less fermentation products, a finding which agrees with experimental data. Complex I of the electron transport chain was to be present in *M. pulcherrima*, but absent in the others. The predicted importance of Complex I was diminished when we incorporated constraints on the amount of enzymatic protein, as this shifts the metabolism towards fermentation.

**Conclusions:**

Our results suggest that Complex I in the electron transport chain is a key differentiator between *Metschnikowia pulcherrima* and the other yeasts considered. Yet, more annotations and experimental data have the potential to improve model quality in order to increase fidelity and confidence in these results. Further experiments should be conducted to confirm the *in vivo* effect of Complex I in *M. pulcherrima* and its respiratory metabolism.

**Supplementary Information:**

The online version contains supplementary material available at 10.1186/s12859-023-05506-7.

## Background

In recent years, there has been increased interest in using alternative non-*Saccharomyces* yeasts for beer and wine brewing [1–7]. In general, there are two primary drivers for the adoption of non-*Saccharomyces* fermentation strains: First, wine producers aim to decrease the resultant alcohol content in their products. Second, some brewers seek to enhance the complexity of aroma compounds, thereby emulating the rich flavor profile of spontaneously fermented beverages. In this study, our primary focus will be on the pursuit of reduced alcohol content.

Climate change has resulted in warmer and sunnier summers in wine producing regions, leading to higher sugar content in ripe grapes. When the must of high sugar grapes are fermented, this leads to higher alcohol content in the product. As a consequence, the alcohol content of wine has risen by approximately 1% alcohol by volume each decade since the 1980s in some wine producing regions [8, 9]. Whereas approaches such as dilution of the must, earlier harvesting of the grapes, and post-fermentation removal of alcohol can bring down the resulting alcohol content, such approaches come at the expense of diminished oenological qualities as well as breaking with established standards for wine brewing [10]. Additionally, these practices may violate local, national, and international regulations, such as the OIV Codex [11, 12]

In order to create wines with reduced alcohol content without losing the rich flavours, aeration during the fermentation process has been proposed as a solution [6]. Unfortunately, using this approach with the canonical wine yeast *Saccharomyces cerevisiae* has proven to be challenging. First of all, the most common strains of *S. cerevisiae* are Crabtree positive, meaning that glucose predominantly gets fermented to ethanol even when oxygen is available [13–15]. Furthermore, aeration often leads to the production of acetic acid, which is considered an undesired by-product [15, 16]. On the other hand, many non-*Saccharomyces* yeasts produce less acetate and are Crabtree negative [2, 17, 18].

Using non-*Saccharomyces* yeasts alone is usually not a good option due to production of bad-tasting compounds and their low tolerance to ethanol. The latter short-coming leads to stuck fermentations and poor wine quality [6, 16]. Experiments attempting simultaneous inoculations of *S. cerevisiae* and non-*Saccharomyces* strains have revealed that exposure of *S. cerevisiae* to oxygen causes unacceptable amounts of acetate production, even after aeration is turned off [16]. In order to mitigate this problem, a technique with sequential inoculation has been developed. In this method, the must is inoculated with the non-*Saccharomyces* yeast with air sparging for two to three days before *S. cerevisiae* is added in order to complete the fermentation. This has proven to be a more viable approach for production of wine with reduced alcohol content, as the production of acetic acid remains low [19–22].

In order to explain and predict such metabolic properties of yeast, genome-scale metabolic models (GEMs) have become a widely used tool [23–29]. For the model organism *S. cerevisiae*, well curated models exist [30, 31] which have been used for a variety of purposes. One application is for the explanation of the Crabtree effect using enzyme constrained genome scale models (ecGEMs) [25, 26, 32]. The ecGEMs incorporate enzymes’ turnover numbers and masses for constraining the internal metabolic fluxes, as the total mass which can be allocated for enzymatic proteins is limited.

In contrast to *S. cerevisiae*, GEMs are not readily available for most non-*Saccharomyces* yeasts. Nevertheless, the development of tools that enable automatic generation of these models from genomic data presents a possible solution to address this limitation. A promising approach, known as ”carving” as described by Machado and coworkers [33], involves generating models from a meticulously curated universal model that serves as a comprehensive database of interconnected biochemical reactions. Furthermore, tools exist also for the incorporation of enzymatic constraints [34, 35] by automatically querying databases for protein masses and turnover numbers in order to integrate these data into an ecGEM.

In this article, we constructed GEMs for five of the most commonly applied non-*Saccharomyces* yeast strains attempted in wine brewing [4, 7, 36]. These are: *Hanseniaspora osmophila*, *Kluveromyces lactis*, *Metschnikowia pulcherrima*, *Torulaspora delbrueckii*, and *Lachancea thermotolerans*. The models were automatically constructed from genome data and carved form a curated universal yeast model [37, 38]. Using the reconstructed GEMs, we predict physiological properties of the yeasts *in silico*.

## Results

### Characteristics and properties of automatically reconstructed genome-scale metabolic models

GEMs of the five non-*Saccharomyces* yeast strains were created by using CarveFungi [37, 38]. From these models, protein constraints were incorporated, and ecGEMs (sMOMENT) were made with AutoPACMEN [34] (see Methods for details). Key properties of the models are summarized in Table 1. In addition, we conducted automated quality checks of the models using MEMOTE [39], and we provide summarized findings in Table 2. From this summary, we observed that the results are similar for all the CarveFungi models. The major shortcomings discovered by MEMOTE was that genes, reactions, and metabolites had few database annotations and identifiers. Also, the MEMOTE check declared the models were stoichometrically inconsistent.

**Fig. 1:**
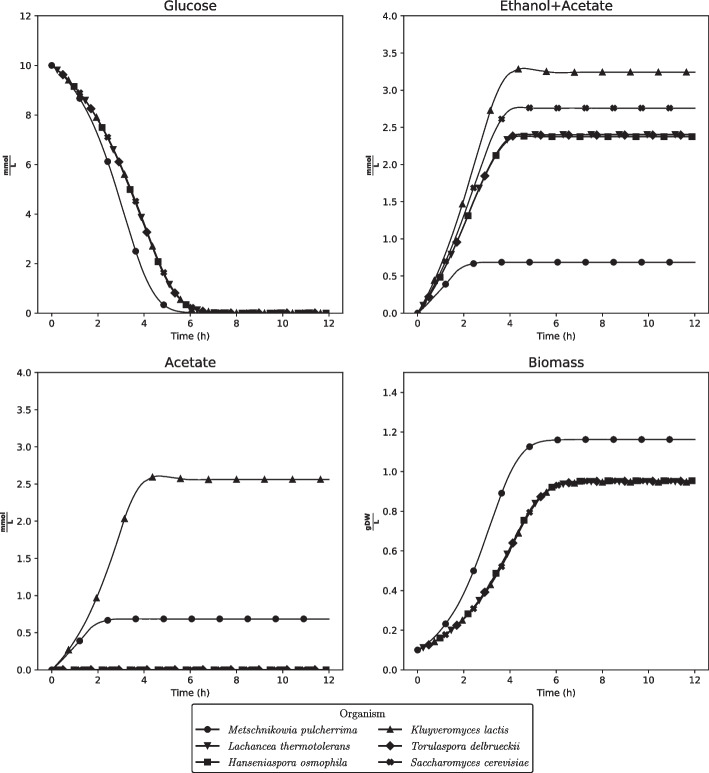
dFBA simulations of the models without enzymatic constraints for the six yeast models, starting with $10\,{\hbox {mmol}}\,\, {\hbox {L}}^{-1}$ glucose. $\frac{\textrm{gDW}}{\textrm{L}}$: Grams of dry weight per liter

We begin our investigation of the models’ phenotypic properties by predicting batch culture growth using dynamic FBA (dFBA) [40] simulations for 12 h. We first use models without enzymatic constraints (Fig. 1). As a reference, we include the *S. cerevisiae* model iND750 [41]. Even though more modern and extensive models of *S. cerevisiae* exist [30], we chose iND750 as it has a comparable size and complexity to the models generated by CarveFungi. However, in the development of the CarveFungi pipeline, the reconstructed yeast was compared with the latest consensus yeast model [37, 38]. We choose glucose as the sole carbon source, with the initial concentration set to $10\,\, {\hbox {mmol}}\,\,{\hbox {L}}^{-1}$ $(1.8 \,\,{\hbox {g}}\,\, {\hbox {L}}^{-1})$. The supply of oxygen was restricted to 10  mmol/g DW Biomass/h (corresponds to 180 mg/g WD / h). See Methods for uptake kinetics and additional details on the simulations.

We observed that simulation results for *M. pulcherrima* are quite different from the other yeasts, since less fermentation (production of ethanol and acetate) was undertaken compared to the other yeasts, and the growth dynamics resulted in higher biomass yield. We also initiated the simulations with $1000\,\, {\hbox {mmol}}\,\, {\hbox {L}}^{-1}$ ($180\,\, {\hbox {g}}\,\, {\hbox {L}}^{-1}$) glucose, which is a more realistic sugar concentration in grape must used in wine fermentation (Additional file 1: Fig. S1). This resulted in a higher degree of fermentation due to the fact that the balance between glucose and oxygen availability was shifted. Still, the same tendencies of *M. pulcherrima* to produce less fermentation products and attain higher biomass, were evident.

In addition to the glucose concentration, we simulated the effect of the sugar composition of the must. This was conducted by replacing the $10 \,\,{\hbox {mmol}}\,\, {\hbox {L}}^{-1}$ with the equivalent amount of fructose (see Additional file 1: Fig. S4). The same trends which we observed for glucose hold true also when fructose is the carbon source.

For *K. lactis* and *L. thermotolerans* there already existed published GEMS; the models iOD907 [42] and iBM3063 [43]. These two models were assessed individually with their corresponding CarveFungi [37, 38] model in order to compare them. MEMOTE reported a lower quality of iOD907 than for the corresponding CarveFungi model for *K. lactis* (Table 2). In part, the low MEMOTE score for iOD907 was due the fact that the molecular formulas of the metabolites were not written in a standard-compliant manner. *K. lactis* is know for its ability to utilise lactose as a substrate [44], for which reactions were present in both models. Comparing batch growth on glucose for iOD907 and the CarveFungi model of *K. lactis* (Additional file 1: Fig. S2), we find some slight differences. iOD907 preferred to produce ethanol instead of acetate and had a somewhat lower biomass yield. For iBM3063, the model had a similar MEMOTE score to the CarveFungi model of *L. thermotolerans*, but we were unable to obtain physiologically plausible results when running dFBA on the model.

Further, we compared the CarveFungi model of *K. lactis* with published experimental data by Dias et al. [45]. In this experiment, *K. lactis* was kept in a chemostat under different dilution rates with glucose as the carbon source. Uptake and secretion rates of glucose, oxygen, carbon dioxide, and glycerol were measured. We locked the uptake and secretion rates of the *K. lactis* GEM to the measured values and optimized for maximal growth. In order to account for protein constraints, we used the sMOMENT model of *K. lactis* under varying levels of the protein pool. Comparing the results with the experimentally determined dilution rates (which is equal to the rate the organism must grow in a chemostat), we found that the modelled growth rates at infinite protein pool were generally somewhat lower than the experimentally determined ones, but still within the experimental variability (Additional file 1: Fig. S3). From these observations, we made two conclusions: First, we think that internal enzyme constraints were not limiting the growth in the experiments considered. Second, we found the *K. lactis* GEM to accurately predict the growth rate given the observed uptake and secretion rates.

### Complex I differentiates *Metschnikowia pulcherrima* from the other yeast species

Having discovered that the GEMs predicted distinct metabolic phenotypes for *M. pulcherrima* compared to the other yeast strains, we were interested in elucidating the underlying reasons for this difference. While the yeast strains (aside from *M. pulcherrima*) performed similarly, we chose to pick *K. lactis* as a representative for these four strains. This is because, to our knowledge, *K. lactis* is the one strain for which the most research has been conducted. To address the metabolic phenotypes, we conducted a comparative analysis between the metabolic models of *M. pulcherrima* and *K. lactis*, excluding enzymatic constraints from consideration. This approach aimed to identify possible causal factors stemming from variations in metabolic network connectivity.

Directly comparing their reaction content, we found that 191 of the reactions in the *M. pulcherrima* model were not present in the *K. lactis* model. To probe the functional consequence of these reactions, we sequentially (and cumulatively) removed each of these *M. pulcherrima* reactions and optimized for biomass production. The nutrient environment used for this assessment was identical to the one used for initiating the dFBA simulations. Some of the reactions were essential and therefore reinserted into the model before continuing. Of the considered reactions which were not essential, we observed two reactions which altered the growth rate: Complex I in the respiratory electron transport chain (NADH dehydrogenase), and mitochondrial Methylenetetrahydrofolate dehydrogenase (NAD+). Note that, removal of Complex I alone was sufficient to produce the same growth as in *K. lactis*. Conversely, adding the Complex I reaction to the *K. lactis* model yielded the same growth rate as of *M. pulcherrima*. Not surprisingly, removal of mitochondrial Methylenetetrahydrofolate dehydrogenase (NAD+) from *M. pulcherrima* did not have any effect on its own, nor did addition of the same reaction into the model of *K. lactis*: Since Complex I pumps protons and the mitochondrial Methylenetetrahydrofolate dehydrogenase does not, Complex I is beneficial when growth is APT dependent. Therefore, we chose to focus on Complex I when further comparing the models.

According to the reconstructions, *M. pulcherrima* was annotated with Complex I, whereas none of the other yeast strains contain this reaction. *Saccharomyces*, *Kluveromyces*, *Torulaspora*, *Lachancea*, and many other yeasts do not have the canonical Complex I of the electron transport chain, but instead feature an alternative Type II NADH dehydrogenases which does not pump protons across the mitochondrial membrane [46–48]. According to the GEM, Complex I pumps 4 protons across the mitochondrial membrane for each molecule of NADH being reduced, whereas the alternative Type II NADH dehydrogenases do not possess this ability. Hence, *M. pulcherrima* is able to create a larger proton-motive force (PMF) per mole of NADH being oxidized, which in turn increases the efficiency in generation of ATP per mole of glucose.

In order to obtain further evidence that Complex I was indeed present in *M. pulcherrima*, we conducted a BLAST [49] search with the protein sequences of *M. pulcherrima* against proteins annotated with Complex I functionality (EC number 7.1.1.2). This search was conducted as a blastp search through UniProt’s web portal [50] using standard settings. Our query returned matches to three manually curated Complex I subunits in Swiss-Prot [50], all for *Neurospora crassa* with evidence on transcript level: NADH-ubiquinone oxidoreductase 19.3 kDa subunit, mitochondrial; NADH-ubiquinone oxidoreductase 23 kDa subunit, mitochondrial; and NADH-ubiquinone oxidoreductase 24 kDa subunit, mitochondrial. The similarity to these sequences were (with corresponding *E*-value): $82\%$ ($2.8\times 10^{-106}$), $73.4\%$ ($1.6 \times 10^{-109}$), and $53.9\%$ ($1.5\times 10^{-84}$), respectively.

**Fig. 2:**
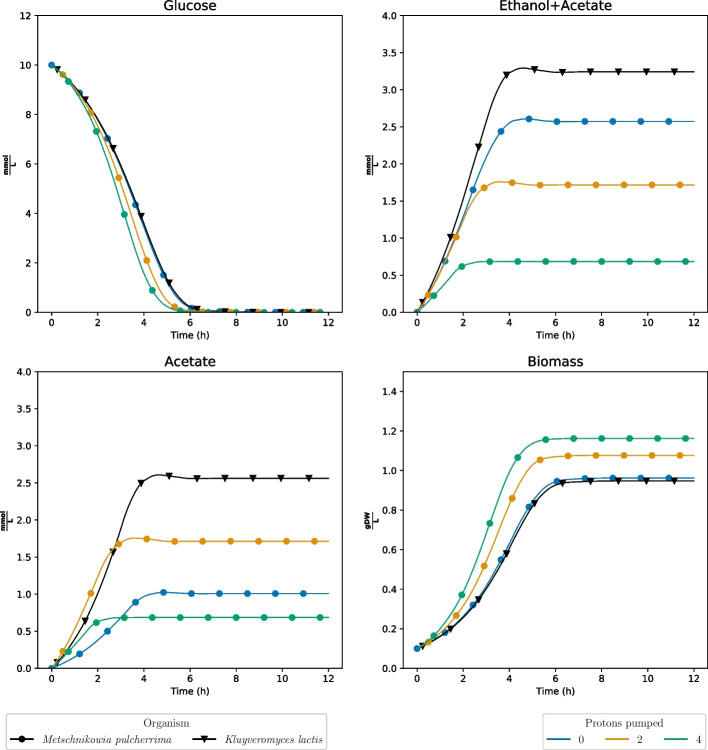
dFBA simulations of the models without enzymatic constraints for *Metschnikowia pulcherrima* and *Kluyveromyces lactis* when artificially changing the stoichiometry of the number of protons pumped by Complex I

In light of these discoveries, we suspected that by removing the advantage of proton pumping in Complex I, the metabolism of *M. pulcherrima* would become more similar to that of the other yeast strains. We therefore artificially changed the stoichiometry of the reaction to two or zero protons being pumped for each molecule of NADH consumed. We conducted a new set of dFBA simulations without enzyme constraints and with the same starting conditions as earlier, using *K. lactis* (lacking Complex I) as a baseline (Fig. 2). From these results, we observe that the glucose consumption and biomass production are more or less identical for *M. pulcherrima* and *K. lactis* when the proton pumping is turned off. These results were identical to that of knocking out Complex I completely. Additionally, in the case of the partially inhibited state where two protons are pumped, the biomass yield and glucose consumption exhibit intermediary values, positioned between those observed in the wild-type and fully inhibited states. For the production of ethanol and acetate, it was observed that the production of ethanol and acetate decreased with the number of protons pumped by Complex I, yet *K. lactis* still displayed a higher production of ethanol and acetate when no protons were pumped.

### Two reactions explain reduced production of fermentation products from *M. pulcherrima* in absence of Complex I activity

*K. lactis* exhibited a greater production of fermentation byproducts (acetate and ethanol) compared to *M. pulcherrima* even in the presence of inactive Complex I. This observation suggested the involvement of supplementary reactions. To explore this hypothesis, we deactivated Complex I in *M. pulcherrima* and performed cumulative knockouts of the reactions exclusive to this organism. We then assessed the combined production of acetate and ethanol following each knockout event to elucidate the potential role of these unique reactions in the observed differences. By this strategy, we found two reactions accounting for the difference in fermentation products. These two reactions were mitochondrial L-glutamate:NADP+ oxidoreductase (Eq. 1) and cytosolic Isocitrate:NADP+ oxidoreductase (Eq. 2), running in the directions illustrated by the equations:

1 $$\begin{aligned} {\hbox {AKG}} + {\hbox {H}}^{+} + {\hbox {NADPH}} + {\hbox {NH}}_{3} \longrightarrow {\hbox {GLU}} + {\hbox {H}}_{2}{\hbox {O}} + {\hbox {NADP}}^{+}, \end{aligned}$$

2 $$\begin{aligned} {\hbox {ICIT}} + {\hbox {NADP}}^{+} \longrightarrow {\hbox {AKG}} + {\hbox {CO}}_{2} + \hbox {NADPH}, \end{aligned}$$

where AKG is $\alpha$-ketoglutarate, GLU is L-glutamate, and ICIT is Isocitrate. The reactions are complementary, meaning that both reactions had to be removed in order to observe an increase in fermentation products (sum of ethanol and acetate). Conversely, adding either of these two reactions to the model of *K. lactis* resulted in a decrease in fermentation products.

None of the models have any reaction transporting $\alpha$-ketoglutarate from the mitochondrion to cytosol directly. For *K. lactis*, reaction (2) occurs in the mitochondrion only, and reaction (1) occurs in only in the cytosol. On the other hand, both of reactions (2) and 1) can occur in either of the two compartments in *M. pulcherrima*. Hence, *M. pulcherrima* obtains a metabolic advantage by being able to carry out both reactions in the same compartment.

We also conducted dFBA simulations where these two reactions in *M. pulcherrima* were knocked out (Additional file 1: Fig. S5). We found growth, glucose consumption, and total fermentation to be almost identical in the two models when Complex I was left inoperative. Likewise, there were no major difference between the models when Complex I was active. Subsequently, the net effect of being able to carry out both reactions in the same compartment is most likely negligible.

### Protein constraints result in changes in use of metabolic pathways

Considering that Complex I is a key differentiator for *M. pulcherrima* with infinite amounts of enzymatic protein available, we next studied how the activity of Complex I affected metabolism when the available enzyme pool was constrained.

**Fig. 3:**
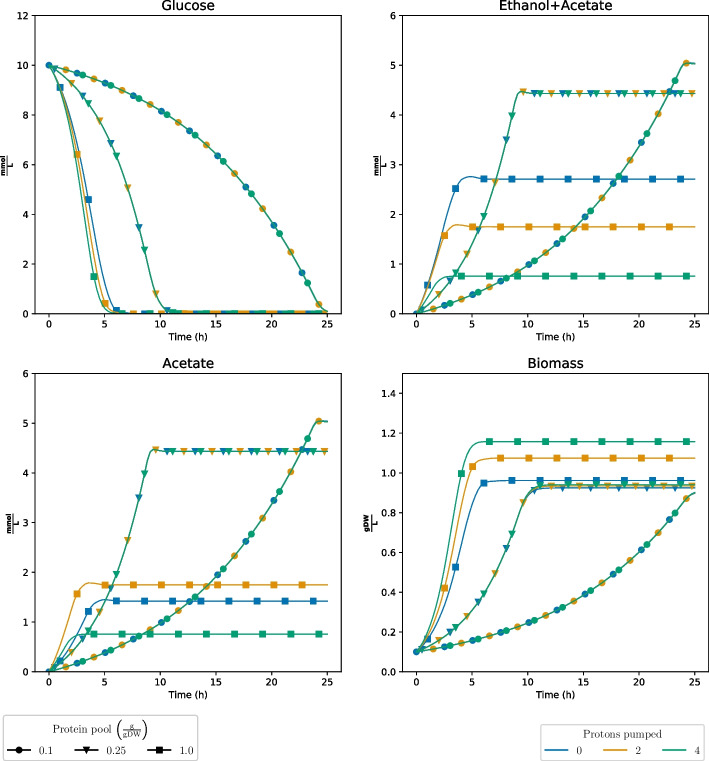
decFBA simulations of the sMOMENT model of *Metschnikowia pulcherrima* when artificially changing the stoichiometry of the number of protons pumped by Complex I under different levels of the protein pool. $\frac{\textrm{gDW}}{\textrm{L}}$: Grams of dry weight per liter

When simulating the effect of Complex I stoichiometry of *M. pulcherrima* with dynamic enzyme constrained FBA (decFBA) [26, 51] (Fig. 3), we could not observe any major effect of the stoichiometry of Complex I for low availability of enzymatic protein. However, at the highest chosen protein pool level, the biomass yield was higher the more protons were pumped by Complex I. This is as expected, since decFBA becomes equivalent to dFBA when the available enzyme pool approaches infinity. For intermediate levels of the protein pool, the effects of stoichiometry were marginal. Most likely, this means that other metabolic pathways are chosen when availability of enzymatic protein is scarce, and hence, is not reliant on Complex I to the same degree. Moreover, the production of acetate and ethanol was affected by the number of protons pumped at the highest level of the enzyme pool only.

**Fig. 4:**
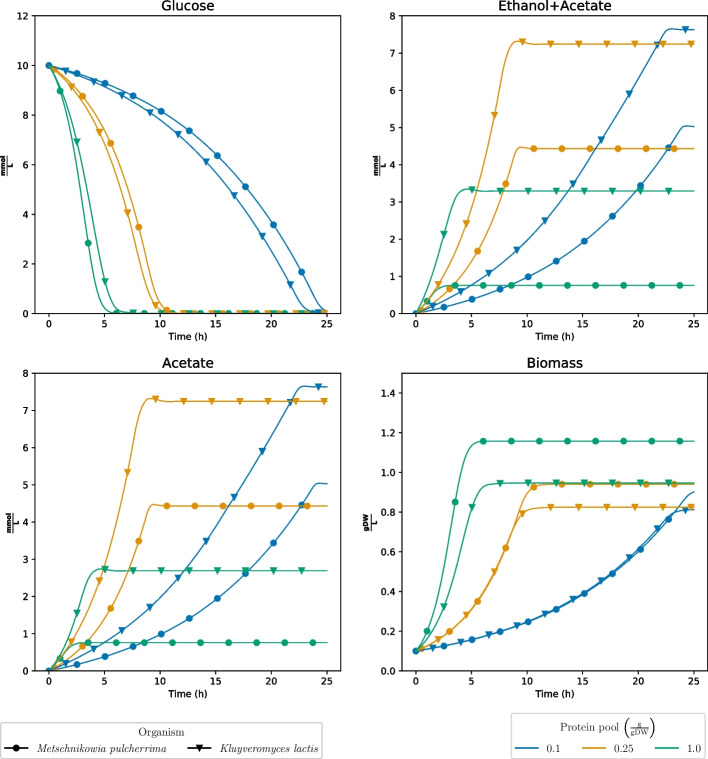
decFBA simulations of the sMOMENT models for *Metschnikowia pulcherrima* and *Kluyveromyces lactis* under different levels of the protein pool. $\frac{\textrm{gDW}}{\textrm{L}}$: Grams of dry weight per liter

Finally, we compared the sMOMENT models of *M. pulcherrima* and *K. lactis* as to get an overview of how the species compare when the access to enzymatic protein was restricted (Fig. 4). As expected, the growth rate increased with the protein availability, but only up to a certain point where the substrate uptake rates became limiting. Also, the results show that the growth rate and biomass yield for *M. pulcherrima* was higher than for *K. lactis* at high availability of enzymatic protein. For lower levels of the enzyme pool, the two strains grew almost equally fast until glucose was exhausted. However, the biomass yield was marginally higher for *M. pulcherrima* than for *K. lactis*, even at the low level of protein, an observation which is due to differences in $k_{cat}$ values in the two models. In addition, *M. pulcherrima* produced less acetate than *K. lactis* for all levels of the enzyme pool. Respiration is energetically more efficient than fermentation in utilization of the carbon source, but comes with a higher protein cost per unit of ATP produced [25, 32]. For this reason, we would expect to observe fermentation at high levels of enzymatic protein, in agreement with our observations. For the lowest level of enzymatic protein, Complex I is less relevant, but the difference in fermentation products can still be explained by the presence of L-glutamate:NADP+ oxidoreductase and Isocitrate:NADP+ oxidoreductase as we discussed earlier.

## Discussion

In our metabolic model reconstructions, we found the GEM of *M. pulcherrima* to provide quantitatively different phenotypes compared to the other models, as it utilized glucose more efficiently, had a higher biomass yield, and conducted less fermentation. To a great extent, this echoes recent research which suggest *M. pulcherrima* as a good candidate for reducing alcohol content in wine [16, 19, 21, 22, 36, 52]. The connection between the three observed effects are quite straight forward. Respiration, instead of fermentation, gives better energy utilization of the substrate, less fermentation products and better growth for the same amount of substrate consumed.

Nonetheless, for certain yeast species, possessing an extensive respiratory metabolism may not confer an evolutionary advantage for two primary reasons. Firstly, the capacity to respire may not be advantageous in environments where oxygen supply is insufficient to sustain full respiration, and the production of ethanol inhibits competitors, as observed in commercial wine fermentation tanks [47, 53]. Secondly, in conditions characterized by high glucose concentrations, yeast may achieve elevated ATP production flux through ethanol fermentation as opposed to respiration, given that the latter necessitates greater protein utilization than the former [25, 26, 32, 54].

Our results suggest that the presumed presence of Complex I in *M. pulcherrima* allows the organism to respire glucose more efficiently than the other yeast strains. Thus, *M. pulcherrima* may be better adopted for respiration and will, therefore, prefer this mode of metabolism. Likewise, Malina et al. [25] attributed the high biomass yield and low production of ethanol and acetate of *Kluyveromyces marxianus* compared to other yeast strains, to be due to its presence of Complex I. At least for some obligate aerobic yeasts, such as *Yarrowia lipolytica*, *Rhodotorula muciluginosa*, and *Candida silvae*, Complex I is present, and inhibiting its activity [48, 55] leads to a reduction in respiration. According to Büschges et al., the yeasts with Complex I were suspected to use alternative NADH dehydrogenases when Complex I was inhibited, albeit with a penalty in growth, just as we observed with *M. pulcherrima*.

The sMOMENT models had shortcomings related to the enzyme constraints. First, CarveFungi included reactions which were not annotated with a specific gene, meaning that these reactions in the downstream sMOMENT models, did not draw from the protein pool but were available without a protein cost. In particular, the mitochondrial genome was not sequenced for the non-*Saccharomyces* strains, meaning that the protein cost of some electron transport chain reactions were far from realistic values. Second, several metabolic enzymes exist as multimers, yet we did not have any information on the subunit stoichiometry. As a result, AutoPACMEN considered that exactly one of each distinct subunit was present in each complex. Furthermore, database $k_{cat}$ values have been shown to be variable and often far from realistic *in vivo* values [56–58]. Finally, the $k_{cat}$ coverage for non-model organisms is low. In our case, the databases contained only $k_{cat}$ entries at the same genus level for *L. lactis*, there were 11 reactions for *K. lactis* ifself, and 5 reactions for *Kluyveromyces marxianus*. For the other non-*Saccharomyces* strains, no $k_{cat}$ data was available within the same genus. This meant that *S. cerevisiae* was the closest available candidate for picking $k_{cat}$ values in many cases. We consider novel approaches for inferring *in vivo* $k_{cat}$ from large-scale experimental data to be the best option for parameterizing high-quality models, although producing the data will be expensive an labour-intensive [56, 57, 59].

Our choice of parameters for dFBA simulations were based on educated guesses in lack of good data for calibration. Sànchez et al. used a enzymatic protein pool of $P_{tot}={0.448}{\hbox {g}} / {\hbox {gDW}}$ and saturation factor of $\sigma =0.5$ for their ecYeast7 model of *S. cerevisiae*. In our case, this would correspond to a simulated protein pool of approximately 0.22 g/gDW since we assumed full saturation. Still, the protein cost of some enzymatic reactions were not accounted for in the sMOMENT models, so we think a somewhat lower enzyme pool would make a fairer comparison. Glucose uptake rates have been shown to vary considerably between different species of yeast and even between different strains of *S. cerevisiae* [60, 61]. From the available data and literature [62], we consider our chosen parameters to be within a realistic range. We believe that applying these values for all organisms would make the most unbiased comparisons given our lack of data even it is possible that the major *in-vivo* differences between the species are caused by variations in their carbon uptake rates.

Nevertheless, we acknowledge that glucose uptake and its balance to oxygen uptake is crucial to the nature of the fermentation. Less oxygen available compared to the consumption of glucose will favour fermentation at the expense of respiration. Additionally, regulatory mechanisms not accounted for by our models most likely also regulate the switching between fermentation and respiration [63]. Comparing Fig. 1 and Additional file 1: Fig. S1, we observed that the glucose concentration has a large effect on the production of fermented compounds, yet *M. pulcherrima* still has a stronger respiratory metabolism than the other yeasts for high glucose concentrations. We did not account for the fact that supplying oxygen is harder when the biomass concentration is high, making a fixed oxygen uptake of 10   mmol/gDW realistic in Fig. 1, but unrealistic in Additional file 1: Fig. S1.

With respect to the sugar composition, we predicted similar growth on fructose as on glucose, assuming that the uptakes rates were equal to that for glucose. Yeast is known to show diauxic growth, meaning that the easiest substrate to degrade is consumed before less favorable substrates are used [64, 65]. Realistic wine must contains both glucose and fructose. Research has suggested that *S. cerevisiae* has a stronger tendency to utilize glucose as compared to fructose, leading to a higher residual concentration of fructose [66]. Such preferences cannot be modelled with baseline FBA, but we assume that a properly parameterized enzyme constrained model would account for this effect.

Besides uptake parameters and $k_{cat}$ values, reseach has also shown that the formulation of biomass in GEMs can have a large effect on the predictions of the models [67–69]. There is research into systematically determining biomass composition for the purpose of GEMs, but these protocols are still on the experimental stage [70]. We assume that customizing the biomass composition of each of the yeast models to experimental measurement have the potential to improve model fiedelity as soon as these data become available..

The model predictions for *M. pulcherrima* should inspire to further research and investigations into the industrial applications of respiratory yeasts. One of the central questions is whether our claim that *M. pulcherrima* has Complex I is correct, and if so, which phenotypic effects this enzyme has. Rotenone is known to be an inhibitor of Complex I and would therefore be a useful tool to study the activity of Complex I [48, 71, 72]. Systematic studies must be conducted in order to assess how *M. pulcherrima* behaves under varying availability of glucose and oxygen.

## Methods

### Creation of the yeast models

The protein sequences of the five species was obtained from the NCBI database [73–78]. We annotated the function of the proteins with EggNog mapper V2 [79] using Diamond [80] for the search of homologs in the EggNOG ortholog database version 5.

For the automatic model reconstruction, we used the software package CarveFungi [37, 38]. CarveFungi is based on the CarveMe algorithm [33]. CarveFungi creates a score for each reaction in a universal metabolic model by linking their EC numbers to the annotation of the proteins obtained by EggNOG. The software contains a deep learning model to predict the subcellular localization of fungal proteins. This prediction contributes to the reaction score, assigning the reactions to a specific compartment in the model. The reaction scores are then used by a Mixed-Integer Linear Programming problem (MILP) to maximize the reactions present in the universal model with a high score and to minimize the reactions with a low score while maintaining the network connectivity and the model functionality.

The universal metabolic model employed for the reconstruction process was developed by integrating fungal reactions obtained from public databases such as AYbRAH [81], KEGG [82], and MetaCyc [83]. This model was subsequently subjected to manual curation using relevant literature to ensure atom balance and simulatability, accomplished by incorporating exchange reactions and extending the biomass reaction based on the yeast consensus model [30]. A comprehensive description and explanation of the model construction process is available in Refs. [37].

The automated metabolic model reconstruction generated ensembles comprising up to 25 alternative models, each derived from the same genome. For the purpose of our analysis, we consolidated each ensemble into a single consensus model by including a reaction if it appeared in at least half of the models within the ensemble.

### Incorporation of enzymatic constraints

sMOMENT models with enzyme constraints were generated by feeding the GEMs into AutoPACMEN [34] version 0.6.2, applying default parameters. The BiGG metabolite file used by AutoPACMEN was retrieved from the BiGG [84] website (http://bigg.ucsd.edu/data_access, July 2023), while the BRENDA data was downloaded from the BRENDA [85] website (https://www.brenda-enzymes.org/download.php, July 2023). Before providing the models to AutoPACMEN, the models are augmented by Uniprot identifiers using Uniprot’s API. AutoPACMEN retrieved $k_{cat}$ values from SABIO-RK [86, 87] and protein masses from Uniprot [50] using its built-in API interface (July 2023). AutoPACMEN’s model calibrator was not used.

### dFBA and decFBA simulations

The models of the five non-*Saccharomyces* strains and the iND750 *S. cerevisiae* model [41] were simulated *in silico* with dynamic FBA (dFBA) [26, 40]. The COBRApy package (version 0.26.3) [88] was used to handle the models, MEMOTE version 0.13.0 was applied for quality checking the models, and the resulting LP problems were solved by the Gurobi optimizer (version 10.0.2).

Glucose was the sole carbon source available with a maximum uptake flux determined by Michaelis-Menten kinetics: $v_{glc} \le \frac{V_{max,glc}\left[ \textrm{glc}\right] }{K_{M,glc}+\left[ \textrm{glc}\right] }$, where the maximal uptake rate $V_{max,glc}= 10\,\, {\hbox {mmol}}/{\hbox {gDW}}$, the half-saturation constant $K_{M,glc} = 5\,\,{\hbox {mmol}}$, and $[\textrm{glc}]$ was the glucose concentration in the medium which was initiated to $[\textrm{glc}]_0 = 10\,\, {\hbox {mmol}}\,\, {\hbox {L}}^{-1}$ for all simulations expect for Additional file 1: Fig. S1 where $[\textrm{glc}]_0 = 1000\,\, {\hbox {mmol}}\,\, {\hbox {L}}^{-1}$. When simulation were run with fructose instead of glucose as the carbon source, the intial fructose concentration was initiated to $[\textrm{fru}]_0 = 10\,\, {\hbox {mmol}}\,\, {\hbox {L}}^{-1}$ and the same uptake parameters as for glucose were applied.

**Table 1:** Properties of the GEMs studied in this paper

Organism	Origin	Reactions	Reversible reactions	Metabolites	Reactions drawing from protein pool
*Metschnikowia pulcherrima*	CarveFungi	2049	610	1633	1310
*Lachancea thermotolerans*	CarveFungi	2049	618	1647	1319
*Torulaspora delbrueckii*	CarveFungi	1876	559	1510	1163
*Kluyveromyces lactis*	CarveFungi	2131	621	1774	1401
*Hanseniaspora osmophila*	CarveFungi	1556	520	1218	902
*Saccharomyces cerevisiae*	iND750	1266	436	1059	0
*Kluyveromyces lactis*	iOD907	2180	894	2338	0
*Lachancea thermotolerans*	iBM3063	3063	1200	2741	0

sMOMENT versions of the models have more reactions than the listed numbers, as autoPACMEN adds auxiliary reactions and splits reactions drawing from the protein pool into separate forward and backward reactions. The models iND750, iOD907 and iBM3063 were taken from external sources [41–43] and did not have any corresponding enzyme constrained model

**Table 2:** MEMOTE quality summaries for various sections

Organism	Origin	Con.	Metabolite an.	Reaction an.	Gene an.	SBO term con.	Overall
*Metschnikowia pulcherrima*	CarveFungi	51	60	57	40	89	67
*Lachancea thermotolerans*	CarveFungi	52	60	56	40	88	67
*Torulaspora delbrueckii*	CarveFungi	52	60	56	40	88	67
*Kluyveromyces lactis*	CarveFungi	52	60	55	40	88	67
*Hanseniaspora osmophila*	CarveFungi	52	61	56	40	88	67
*Saccharomyces cerevisiae*	iND750	97	80	83	43	82	86
*Kluyveromyces lactis*	iOD907	22	25	25	0	0	12
*Lachancea thermotolerans*	iBM3063	50	74	66	33	88	67

The numbers are given in terms of the percentage of the total achievable score (higher is better and maximum is 100). *Con.* Consistency, *an* annotation

The biomass concentration was as initiated to $\left[ \textrm{X}\right] _0 = 0.1 {\hbox {gDW}}/{\hbox {L}}$. Oxygen was available at a fixed rate of $v_{\textrm{oxygen}} \le 10\,\, {\hbox {mmol}}/{\hbox {gDW}}$.

We monitored several entities, including biomass, glucose, acetate, ethanol, and glycerol. The latter three components were incorporated to track the accumulation of fermentation products generated by the yeast. However, under the tested conditions, none of the models produced glycerol; hence, we omitted its depiction for clarity. Furthermore, we sought to highlight the combined production of ethanol and acetate. Consequently, the graphical representations included a panel displaying the sum of ethanol and acetate concentrations in the medium, and another panel illustrating the acetate concentration separately. To preclude physiologically implausible metabolic exports, we blocked the export reactions for lactate (both stereoisomers), dihydroxyacetone, D-ribulose, arabinitol, and ribose. Additionally, we inhibited a wasteful mitochondrial membrane proton leakage reaction, which would have led to physiologically unreasonable outcomes if not removed from the model.

To obtain consistent and physiologically plausible results, we applied lexicographic objectives when performing FBA on the models prioritized in the following order:

1. Maximize production of biomass
2. Minimize consumption of glucose
3. Maximize excretion of ethanol
4. Maximize excretion of acetate
5. Maximize excretion of glycerol

The models were simulated using the static optimization approach and SciPy’s solve_ivp function [89]. For the ODE solver, the BDF algorithm [90] was used with an absolute and relative tolerance of $10^{-2}$. In cases where the optimization problem became infeasible, the simulation was terminated, but results were padded such that the final state of the system was imputed to all time-points beyond the termination. This happened only if the model was unable to grow because the carbon source (glucose) in the medium was depleted.

dFBA was performed both for the original models generated with CarveFungi and the sMOMENT models processed through AutoPACMEN. Upon running decFBA with the sMOMENT models, the level of the enzyme pool was adjusted. Three different levels of the enzymatic protein pool (0.1, 0.25, and 1.0 grams of protein per gram dry weight(g/gWD)) were chosen.

### Supplementary Information

**Additional file 1**. contains five figures showing supplementary computer simulations and comparisons.

## Data Availability

The source code and data for generating, augmenting and analysing the GEMs used in this paper in addition to the resulting GEMs is available at Figshare (http://dx.doi.org/10.6084/m9.figshare.22664848).

## Abbreviations

OIV: International Organisation of Vine and Wine
FBA: Flux balance analysis
dFBA: Dynamic flux balance analysis
decFBA: Dynamic Enzyme-Constrained FBA
MILP: Mixed-Integer Linear Programming
GEM: Genome-scale metabolic model
ecGEM: Enzyme constrained genome-scale metabolic model
LP: Linear programming
DW: Dry weight
PMF: Proton motive force
ODE: Ordinary differential equation
