# Supplementary material for: Genome-scale metabolic models reveal determinants of phenotypic differences in non-Saccharomyces yeasts
Source: BMC Bioinformatics. 2023 Nov 21;24:438. doi: 10.1186/s12859-023-05506-7 (PMC10664357; doi:10.1186/s12859-023-05506-7)
Supplement: Supplementary file 1 — Additional file 1. contains five figures showing supplementary computer simulations and comparisons. [file 12859_2023_5506_MOESM1_ESM.pdf]

Supplementary material for "Genome-scale metabolic models reveal determinants of phenotypic differences in non-Saccharomyces yeasts"

Jakob Peder Pettersen, Sandra Castillo, Paula Jouhten, Eivind Almaas

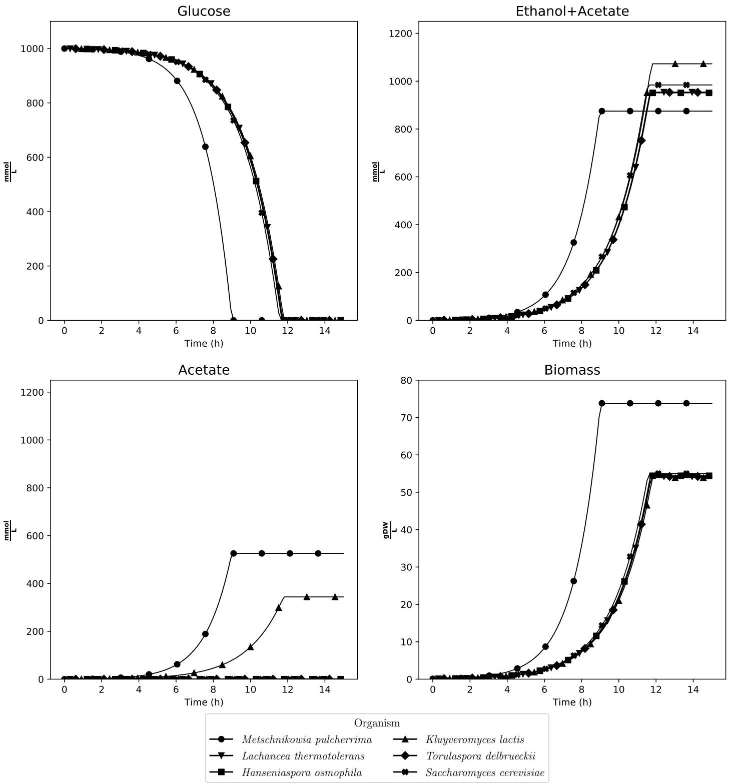

Figure S1: dFBA simulations of the models without enzymatic constraints for the six yeast strains, starting with $1000\,\mathrm{mmol\,L^{-1}}$ glucose. $\frac{\mathrm{gDW}}{\mathrm{L}}$: Grams of dry weight per liter.

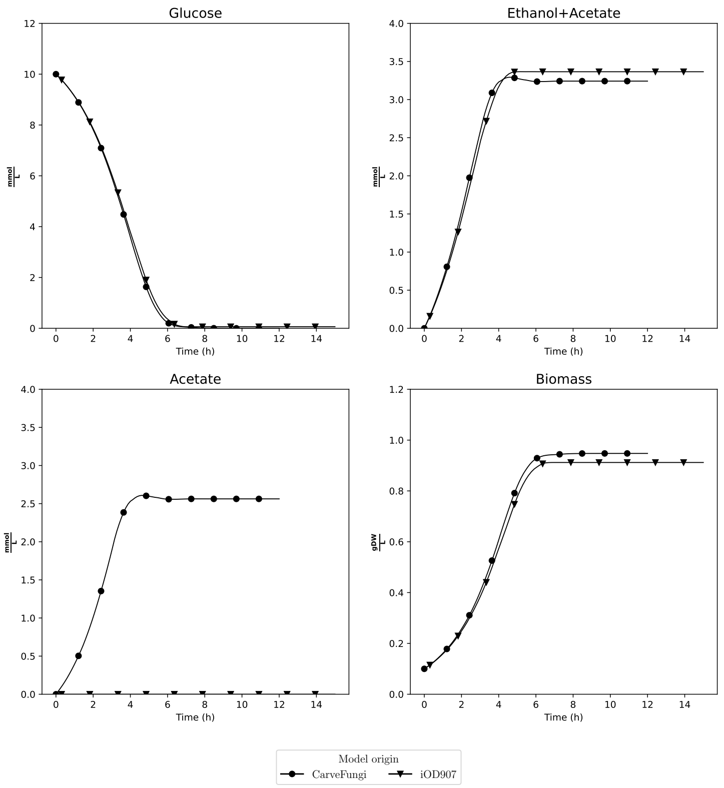

Figure S2: dFBA simulations of the CarveFungi and iOD907 models of *K. lacitis* without enzymatic constraints, starting with $10\,\mathrm{mmol\,L^{-1}}$ glucose. $\frac{\mathrm{gDW}}{\mathrm{L}}$: Grams of dry weight per liter.

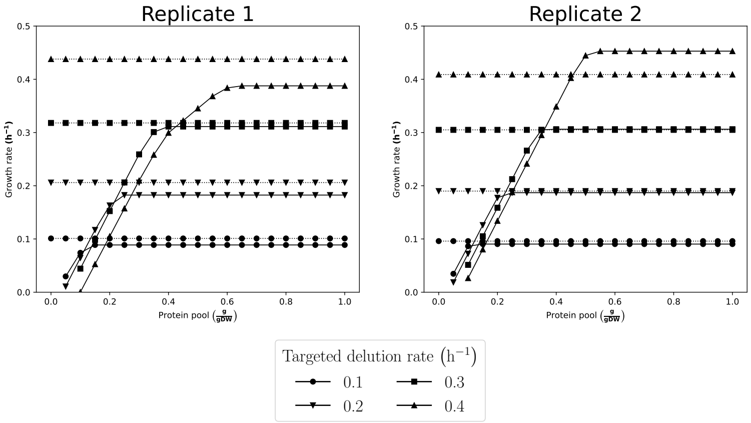

Figure S3: Comparison of the sMOMENT model of *K. lactis* with experimental chemostat data from Dias *et al.* 2018 (see Table 1 of the referenced paper). Each marker shape in each of the two panels corresponds to an sub-experiment of the original data with a correspoinding targeted delution rate of the chemostat. The sMOMENT model has fitted with different levels of the enzymatic protein pool and locked to measured uptake and secretion rates of glucose, oxygen, carbon dioxide and glycerol. Given this constraints, the model was optimized for maximum growth and the growth rate was reported (solid lines). This is compared to the corresponding experimentally determined growth rate marked with the same shape (dotted lines) which was usually somewhat different from the targeted delution rate. $\frac{\mathrm{gDW}}{\mathrm{L}}$: Grams of dry weight per liter.

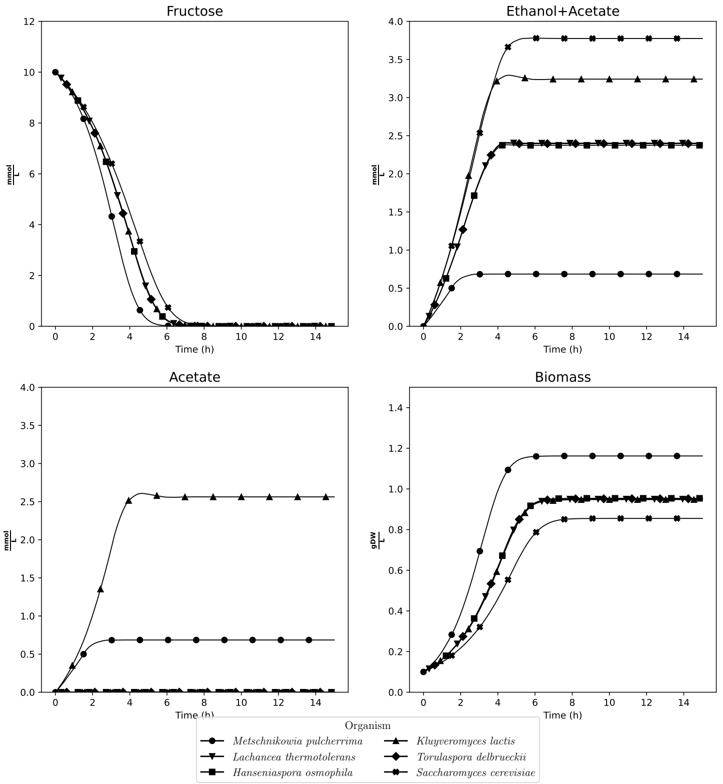

Figure S4: dFBA simulations of the models without enzymatic constraints for the six yeast models, starting with $10\,\mathrm{mmol\,L^{-1}}$ fructose. $\frac{\mathrm{gDW}}{\mathrm{L}}$: Grams of dry weight per liter.

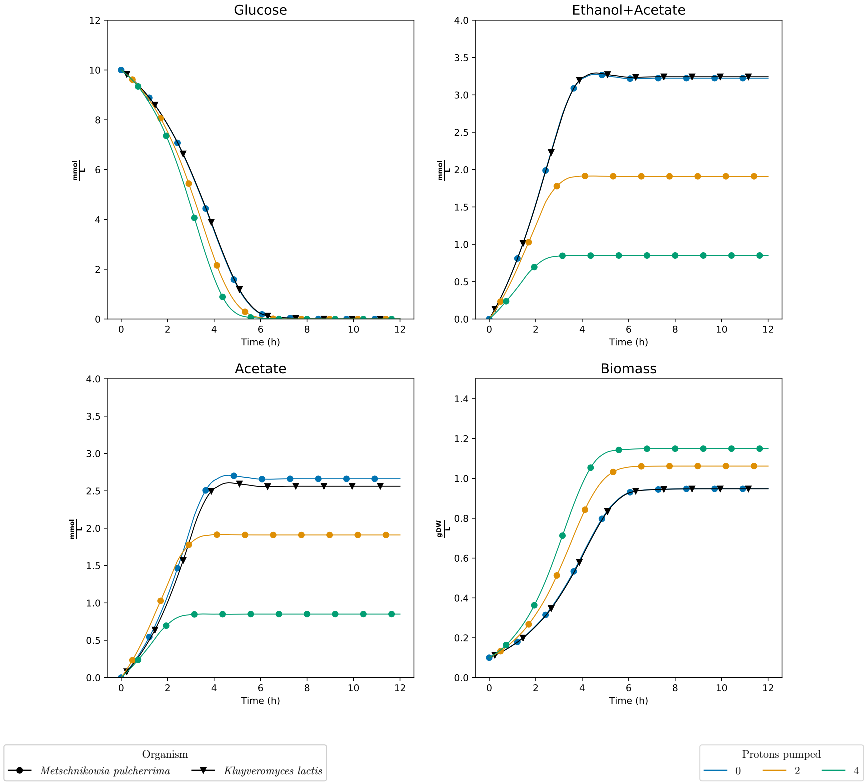

Figure S5: dFBA simulations of the models without enzymatic constraints in *Metschnikowia pulcherrima* and *Kluyveromyces lactis* when artificially changing the stoichiometry of the number of protons pumped by Complex I. In these simulations, the reactions L-glutamate:NADP+ oxidoreductase and Isocitrate:NADP+ oxidoreductase for *Metschnikowia pulcherrima* are knocked out.
